# A scientific note on *Aethina* sp. in wild stingless bee nests

**DOI:** 10.1007/s13592-026-01307-y

**Published:** 2026-09-29

**Authors:** Michael N. K. Muturi, Seunghyun Lee, Seunghwan Lee, Rebecca Denes, H. Michael G. Lattorff, Kiatoko Nkoba, Peter Neumann, Alexis Beaurepaire

**Affiliations:** 1 Institute of Bee Health Vetsuisse Faculty, University of Bern, Schwarzenburgstrasse 161, 3003 Bern, Switzerland; 2 Laboratory of Insect Biosystematics, Division of Entomology, Seoul National University, 1 Gwanak-ro, Gwanak-gu, Seoul, 08826 Republic of Korea; 3 Research Institute of Agriculture and Life Sciences, Seoul National University, 1 Gwanak-ro, Gwanak-gu, Seoul, 08826 Republic of Korea; 4 School of Agriculture and Science, University of KwaZulu-Natal, University Road, Westville, Private Bag X 54001, Durban, 4000 South Africa; 5 International Centre of Insect Physiology and Ecology (icipe), P.O. Box 30772-00100, Nairobi, Kenya; 6 Swiss Bee Research Centre, Agroscope, Schwarzenburgstrasse 161, 3003 Bern, Switzerland

**Keywords:** Parasitology, Population genetics, Host range, Beetle associates, DNA barcoding, Microsatellites

A few beetle species can be found in bee nests, ranging from occasional associates to harmful common parasites (Neumann and Ritter 2004; Krishnan et al. 2015; Camenzind et al. 2024; Wambua et al. 2025). In general, the host ranges of these beetles remain poorly understood, with the notable exception of small hive beetle (SHB), *Aethina tumida*, which is a well-known parasite of both wild and managed honey bee colonies as well as managed stingless bees and bumblebees (Neumann et al. 2016). However, whether any *Aethina* species are associated with wild stingless bee colonies remains unknown. Here, we report for the first time the association of a putative new species of *Aethina* with wild stingless bee colonies.

In February 2024 (local dry season), four underground nests of *Meliponula lendliana* were identified in Kakamega Forest, Kenya (Table S1). The nests were carefully excavated and inspected. Adult beetles (*N* = 71), found in all four nests, were sampled and sexed following standard methods (Neumann et al. 2013). No beetle larvae or eggs were found in any of the stingless bee nests. Samples were preserved in 90% ethanol and stored in a −20 °C freezer until further molecular analysis. The samples were later shipped to the Institute of Bee Health at the University of Bern, Bern, Switzerland. A subset of the samples (three males and three females) was shipped to the Laboratory of Insect Biosystematics, Division of Entomology, Seoul National University, Republic of Korea, for morphological identification.

To conduct the morphological identification, specimens were examined using an Olympus SZ61 stereomicroscope (Olympus Corporation, Tokyo, Japan). Photographs were taken with a Canon EOS 70D digital camera equipped with a Canon MP-E 65 mm f/2.8 1–5× macro lens and controlled using a Cognisys StackShot system. To examine the male and female genitalia and pygidium, specimens were relaxed in distilled water at room temperature for 2 to 4 h. The genitalia were then carefully dissected from the last abdominal segment using hooked pins or fine forceps, while leaving the abdomen intact. The dissected genitalia were macerated in 10% KOH at room temperature for 4 to 6 h and subsequently photographed using a Samsung Galaxy Note S24 Ultra. The body length and thoracic width measurements for the male and female stingless bee beetles were also recorded.

In parallel with the morphological identification, total DNA was extracted from 44 stingless bee beetles using a NucleoSpin® Tissue kit (Macherey-Nagel, Düren**,** Germany). Mitochondrial DNA (mtDNA) was then amplified (*n *= 18 stingless bee beetles) with SHB COI barcoding primers (Evans et al. 2000, Table S2) under optimal thermal conditions (Table S3) and PCR volumes (Table S4). The quality of the PCR products was assessed in a 2% (w/v) agarose gel, cleaned up using the NucleoSpin® Gel & PCR Clean-up kit (Macherey-Nagel, Düren**,** Germany), and sent to the Fasteris Company-Genesupport SA Life Sciences (Plan-les-Ouates, Switzerland) for unidirectional sequencing using the forward primer. To complement the mtDNA data and assess the fine-scale population structure of the beetles, nuclear DNA (nuDNA) from 44 stingless bee beetles was amplified using seven microsatellite primers (Evans et al. 2008; Papach et al. 2025 Table S2) using optimized PCR thermal conditions and reaction volumes (Tables S3, Table S4). PCR products were sent to Microsynth AG (Balgach, Switzerland) for genotyping.

The mtDNA sequences of the stingless bee beetles were first identified using the BLAST software (Altschul et al. 1990). After removing redundant sequences, a final dataset of 63 sequences spanning 985 bp was assembled, consisting of stingless bee beetles, *A. tumida* specimen obtained from nine neighbouring African-derived honey bee colonies in another study (Muturi et al. unpubl. data), and 31 non-*Aethina* nitidulid specimens (Table S5). A second sequence dataset was also constructed using 65 sequences (709 bp long, i.e. shorter than the first dataset) to supplement the first dataset by including two additional *Aethina* sequences: *Aethina aeneipennis* and *Aethina argus* (Table S5). A phylogenetic tree was rooted using Carpophilinae (Table S5) as the outgroup, following the topology of Lee et al. (2025) and using the maximum likelihood method implemented in IQ-TREE (Trifinopoulos et al. 2016). The optimal substitution model (GTR + F + I + G4) was selected using ModelFinder (Kalyaanamoorthy et al. 2017) under the Bayesian information criterion (BIC). Nodal support was assessed using ultrafast bootstrap with 1000 replicates. To determine whether the stingless bee beetles were genetically similar to or distinct from the honey bee beetles, the mtDNA-based genetic distance (p-distance) between the two groups was calculated in MEGA 11 (Tamura et al. 2021). To determine the levels of nuDNA differentiation between the two groups and investigate potential admixture between the beetles, F_*ST*_ values were calculated. As the microsatellite primers used are located on the X chromosome (Papach et al. 2025), the latter analysis was done separately for females (35 stingless bee beetles and 28 honey bee beetles) and males (9 stingless bee beetles and 26 honey bee beetles) in GenAlEx software version 6.5 (Peakall and Smouse 2012). Furthermore, to visualize the genetic distance in the nuDNA between the two beetles, a principal component analysis (PCA) was performed using *R* version 4.4.2 and the *adegenet* package (R Core Team 2024; Jombart 2008).

Detailed morphology revealed that the stingless bee beetles resemble SHB in overall habitus, having an oval, slightly convex body with pale dorsal pubescence and truncated elytra exposing the pygidium. The femora and tarsi are strongly expanded (Figure S1). They can be assigned to the *Aethina*-complex based on the combination of general habitus, a relatively wide distance between the hind coxae, broadly lobed tarsi, and the structure of the genitalia (Lee et al. 2017). However, they differ from *A. tumida* in being smaller, more elongated, and distinctly more parallel-sided (Figure 1). The male genitalia are similar in general structure but differ in having a more parallel-sided tegmen lacking a median constriction compared to that of SHB (Figure S2). Most notably, the new species lacks the characteristic arch-like depression on the pygidium, which is present in *Aethina* s.s. (Figure S3-S4, Kirejtshuk and Lawrence 1999; WOAH (2025); Lee et al. 2017). Both the male and female stingless bee beetles were similar in body length and thoracic width (Table S6).

**Figure 1.**
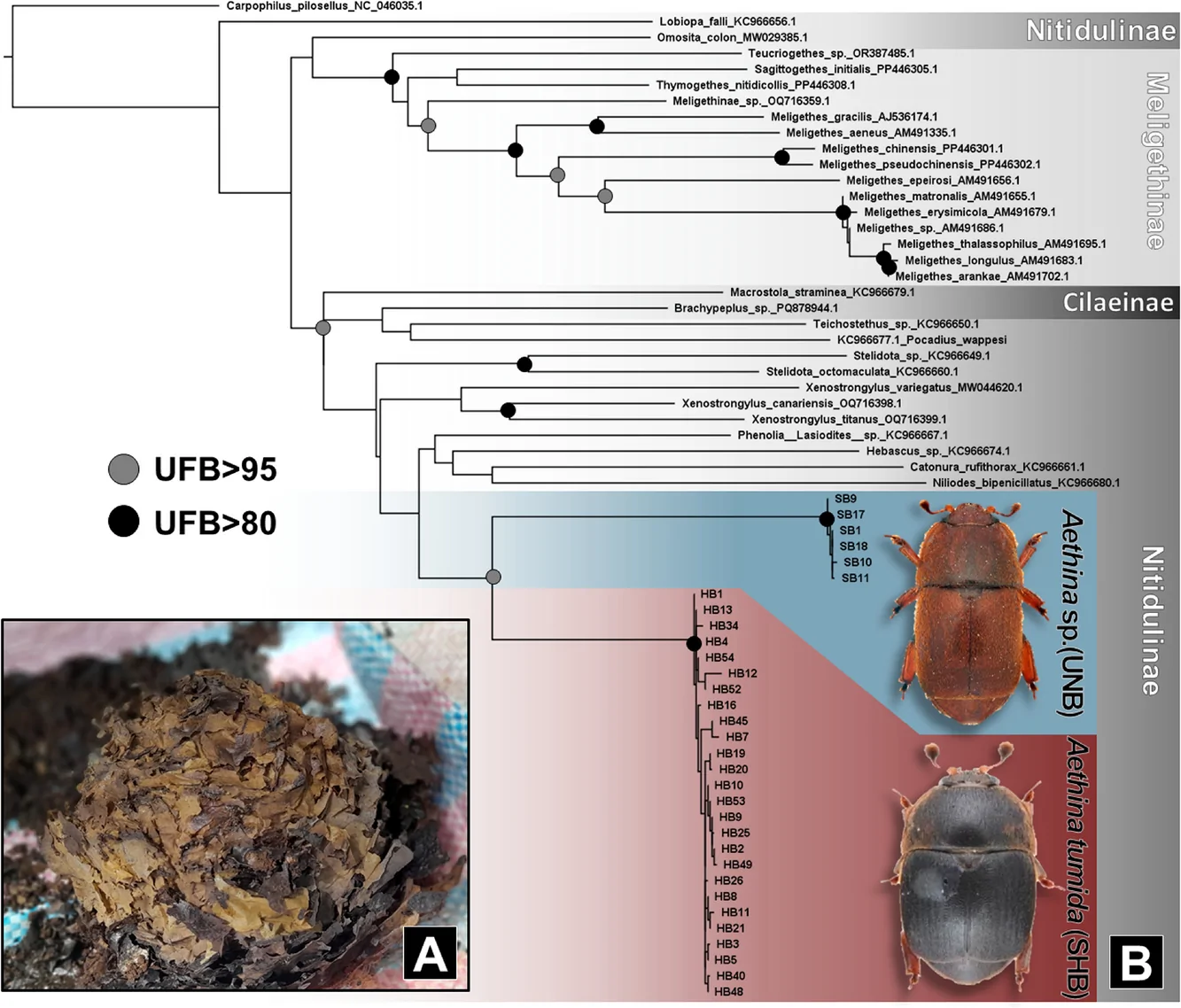
**Comparison of COI sequences of** ***Aethina*** **sp. (underground nest beetle, UNB),** ***Aethina tumida*** **(Small Hive Beetle, SHB), and non-*Aethina*** **nitidulid beetles**. **A** Underground stingless bee nest from which the beetles were sampled. **B** Maximum likelihood phylogenetic tree of COI gene sequences (985 bp) consisting of (i) *Aethina* sp., UNB (six distinct haplotypes); (ii) *A. tumida*, SHB, collected in neighbouring African-derived colonies (27 distinct haplotypes); and (iii) non-*Aethina* beetles from Nitidulinae and Cilaeinae subfamilies obtained from NCBI. Branches are labelled as either grey or black based on their UltraFast bootstrap (UFB) values, which are a quality measure of branch support. The tree shows that UNB are sister taxa to SHB and are different from the rest of the non-*Aethina* beetles.

In both the 985 bp and 709 bp datasets, the *A. tumida* sequences from samples collected in honey bee colonies and sequences from the stingless bee beetles were consistently recovered as sister taxa with an 85%–88% similarity to each other (Figure 1, Figure S5). Both sequence groups differed by 12.4 ± 0.011%, and the same haplotypes were found in males and females. While stingless bee and honey bee beetles were strongly supported as monophyletic, the relationship between the two was only marginally supported. It would be expected that the two available *Aethina* sequences, *Aethina aeneipennis* and *Aethina argus*, would cluster closely to *A. tumida* sequences in the phylogenetic tree as they belong to the same genus. However, this was not the case as the two were distantly separate from *A. tumida*, rendering *Aethina* non-monophyletic. Thus, we report a second COI dataset of 709 bp (Table S5, Figure S5). However, given that *Aethina* was recovered as monophyletic in Lee et al. (2020), this discrepancy in the two datasets is likely attributable to limitations of the COI-only approach used here. Genetic differentiation between stingless bee and honey bee beetles was significant for both sexes (Females F_*ST*_ = 0.265, *p* ≤ 0.001; Males F_*ST*_ = 0.369, *p* ≤ 0.001) and the beetle samples in honey bee and stingless bee colonies clearly segregated, with no hybrid being detected based on the nuclear DNA analysis (Figure 2).

**Figure 2.**
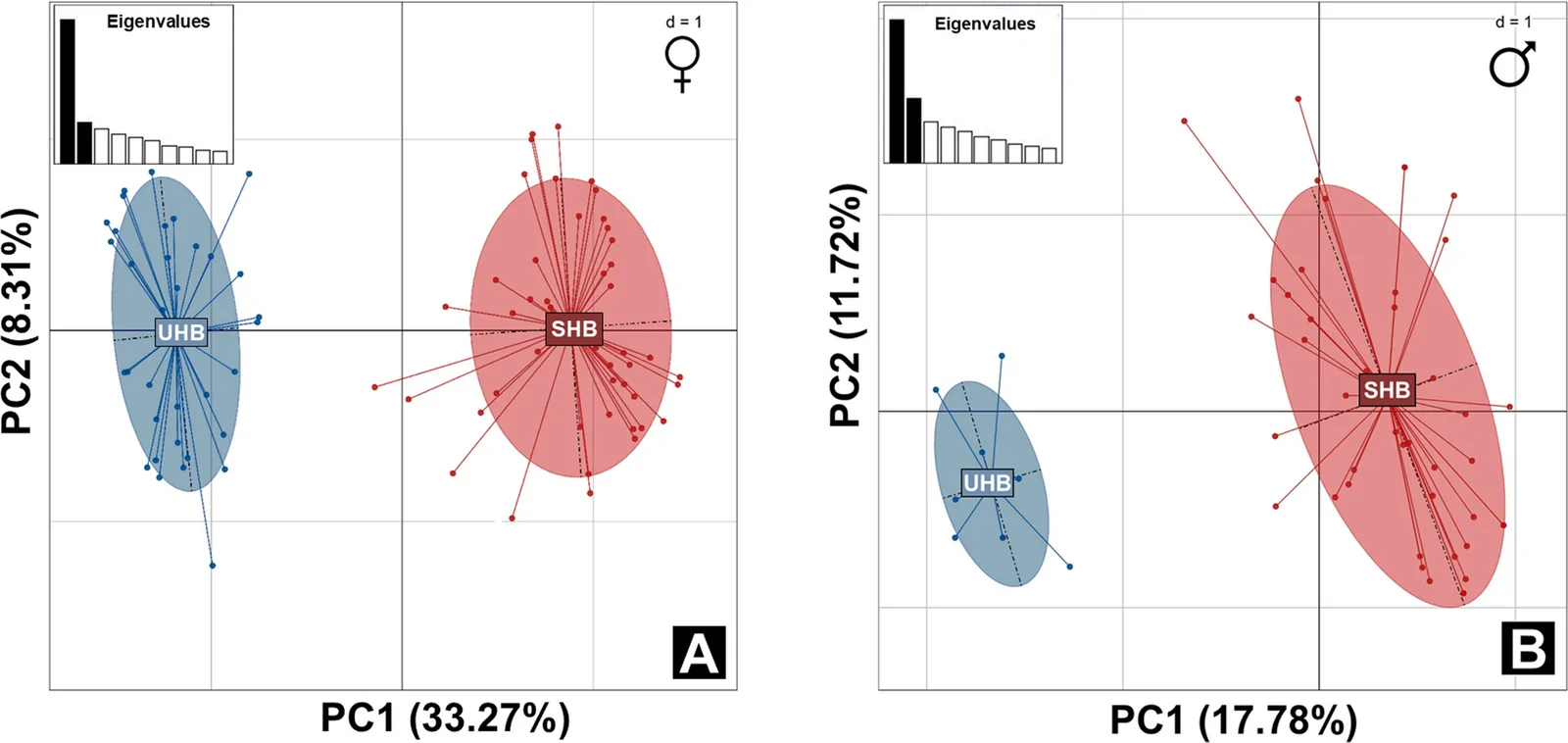
**Genetic structure of underground nest beetle (UNB) and small hive beetle (SHB)**. Principal component analysis (PCA) plot comparing the genetic structure of (**A**) female and (**B**) male beetles from stingless bees*, Aethina* sp. (UNB) and honey bees, *Aethina tumida* (SHB) based on their genotype at seven DNA microsatellite markers. The ellipses represent 95% confidence interval of all individuals sampled from the two populations represented as dots. On the x-axis and y-axis, respectively, are the values of the two principal components that explain the largest amount of variance. Genetic differentiation between UNB and SHB is significant for both sexes (Females F_*ST*_ = 0.265, *p* = 0.001; Males F_*ST*_ = 0.369, *p* = 0.001) and both PCA plots show that the beetles are genetically distinct, as they cluster separately.

Altogether, the combined morphology and genetics data suggest that the beetles sampled in wild stingless bee colonies are a novel species of the genus *Aethina*, proposed here as *Aethina* sp. with its common name, being the underground nest beetle (UNB), a name that has not been previously used. The comparatively large numbers of UNB found in all investigated *M. lendliana* nests indicate a more common association than that observed with other species (e.g., *Cychramus luteus* with *Apis mellifera*; Neumann and Ritter 2004). However, the absence of any beetle eggs or larvae in *M. lendliana* nests needs further investigation to establish the nature of interactions between UNB and its stingless bee host. Notably, it is known that *A. tumida* does not always reproduce in association with *A. mellifera* nests (Neumann et al. 2016), and it is possible that UNB exhibits a similar behaviour. Thus, further investigations are required into the ecology of UNB, its host range across both underground and tree-nesting meliponine species, and its potential impact on its hosts’ health. Further description of UNB is ongoing to prepare for its approval as a new species within the *Aethina* genus.

## Supplementary Information

Below is the link to the electronic supplementary material.

ESM 1 Supplementary Material 1 (DOCX 1.04 MB)

## Data Availability

Sequences will be uploaded on NCBI, and their accession numbers will be provided upon manuscript acceptance.
