# Supplementary material for: A scientific note on Aethina sp. in wild stingless bee nests
Source: Apidologie. 2026 Sep 29;57(5):76. doi: 10.1007/s13592-026-01307-y (PMC13624045; doi:10.1007/s13592-026-01307-y)
Supplement: Supplementary file 1 [file 13592_2026_1307_MOESM1_ESM.docx]

**Table S1:** Colony sites information- Date of sampling, Colony ID, number of male and females beetles sampled from underground *Meliponula lendliana* stingless bee nests (N = 4 nests) and *Apis mellifera* honey bee colonies (N = 9 colonies) in Kakamega forest

| Beetles sampled | Latitude° E | Longitude ° N | Date of sampling | Colony ID | Males | Females | Total |
| --- | --- | --- | --- | --- | --- | --- | --- |
| Stingless bee beetles | 34.854 | 0.288 | 06.02.2024 | 1 | 3 | 11 | 14 |
|  | 34.854 | 0.288 | 06.02.2024 | 2 | 4 | 11 | 15 |
|  | 34.854 | 0.288 | 08.02.2024 | 3 | 2 | 7 | 9 |
|  | 34.854 | 0.288 | 08.02.2024 | 4 | 0 | 6 | 6 |
| Honey bee beetles | 34.963 | 0.243 | 03.02.2024 | 1 | 13 | 24 | 0 |
|  | 34.963 | 0.243 | 03.02.2024 | 2 | 6 | 6 | 0 |
|  | 34.963 | 0.243 | 03.02.2024 | 3 | 7 | 15 | 0 |
|  | 34.952 | 0.231 | 05.02.2024 | 4 | 8 | 14 | 0 |
|  | 34.952 | 0.231 | 05.02.2024 | 5 | 5 | 8 | 2 |
|  | 34.952 | 0.231 | 05.02.2024 | 6 | 17 | 20 | 1 |
|  | 34.938 | 0.264 | 05.02.2024 | 7 | 11 | 12 | 3 |
|  | 34.938 | 0.264 | 05.02.2024 | 8 | 5 | 14 | 13 |
|  | 34.938 | 0.264 | 05.02.2024 | 9 | 4 | 5 | 0 |

**Table S2:** List of primers used in the study. The target DNA region, primers, sequence (5'->3'), product length in bp and references of the primers are shown

| Target | Primer | Sequence (5'->3') | Product length (bp) | Reference |
| --- | --- | --- | --- | --- |
| mtDNA | AT1904S | GGTGGATCTTCAGTTGATTTAGC | 1080 | Evans et al. 2000 |
|  | AT2953A | TCAGCTGGGGGATAAAATTG |  |  |
| nuDNA | Atu-8 | ATGCCAATGCAACAACGTAA | 296 | Papach et al*.* 2023 |
|  |  | TCCTGAACAGTTTCCTGACCT |  |  |
|  | Atu-9 | TCACGACGGCAAGTTTAACA | 287 | Current study |
|  |  | AAACCGTGGACCGTTAATTG |  |  |
|  | Atu-11 | CGGCGTCTTGTGATGATTTA | 281 |  |
|  |  | AAAAACAAACACGCGAATCC |  |  |
|  | Atu-14 | CTTACACATTTCGGGCCATT | 217 | Papach et al*.* 2023 |
|  |  | CCCTTCTCTCAATAGATTGCTTT |  |  |
|  | Atu-16a | CGCCGTTAACACGTAACACA | 118 |  |
|  |  | AGCGACCGCAGTTACTGAAT |  |  |
|  | Atu-17 | CATGATGGTTCGTGAGCAAC | 168 |  |
|  |  | TGGCCACTATAACAGCCAAT |  |  |
|  | Atu-18a | AATACGCATGTTTGCCACTTC | 191 |  |
|  |  | GGGTTTGGCCATCTGAAAAT |  |  |
|  | B89 | CGTCGCTATTAGCCCCTTTA | 110 | Evans et al. 2008 |
|  |  | CTGACCTTCTCGTGCAACAA |  |  |

**Table S3**: PCR thermal conditions used in amplifying the mitochondrial DNA (mtDNA) and nuclear DNA (nuDNA) of both stingless and honeybee beetles

| mtDNA | Step | PCR temperature °C | Time (min) | Cycles |
| --- | --- | --- | --- | --- |
|  | Initial denaturation | 95 | 3 min | 1x |
|  | Denaturation | 95 | 30 s | 35x |
|  | Annealing | 52 | 30 s |  |
|  | Extension | 72 | 70 s |  |
|  | Final extension | 72 | 8 min |  |
| nuDNA | Initial denaturation | 95 | 3 min | 1x |
|  | Denaturation | 95 | 30 s | 35x |
|  | Annealing | 52 | 30 s |  |
|  | Extension | 72 | 70 s |  |
|  | Final extension | 72 | 8 min |  |

**Table S4:** PCR volumes used in amplifying the mitochondrial DNA (mtDNA) and nuclear DNA (nuDNA) of both stingless and honeybee beetles used in the study

| Order | mtDNA | nuDNA |
| --- | --- | --- |
| DNA template | 2 µL | 1 µL |
| water, | 15.9 µL | 6.55 µL |
| 5x My *Taq* reaction buffer | 5 µL | 2 µL |
| Forward primer | 1 µL | 0.2 µL |
| Reverse primer | 1 µL (10 µM) | 0.2 µL |
| *Taq* polymerase | 0.125 µL | _ |
| Total Volume | 25 µL | 10 µL |

**Table S5:** BLAST results blasting of mtDNA sequences of SB beetles. The % similarity and the accession numbers of the closest sequences to SB are also shown

| Sample ID | % similarity | Closest accession no |
| --- | --- | --- |
| SB1 | 88.05 % | [KT380626.1](https://www.ncbi.nlm.nih.gov/nucleotide/KT380626.1?report=genbank&log$=nucltop&blast_rank=1&RID=JV3ACSRZ014) |
| SB2 | 88.13 % | [KT380626.1](https://www.ncbi.nlm.nih.gov/nucleotide/KT380626.1?report=genbank&log$=nucltop&blast_rank=1&RID=JV3CPGUY014) |
| SB3 | 88.07 % | [KT380626.1](https://www.ncbi.nlm.nih.gov/nucleotide/KT380626.1?report=genbank&log$=nucltop&blast_rank=1&RID=JV3FXTHF014) |
| SB4 | 88.04 % | [KT380626.1](https://www.ncbi.nlm.nih.gov/nucleotide/KT380626.1?report=genbank&log$=nucltop&blast_rank=1&RID=JV3JHZDY014) |
| SB5 | 86.73 % | [PQ878931.1](https://www.ncbi.nlm.nih.gov/nucleotide/PQ878931.1?report=genbank&log$=nucltop&blast_rank=1&RID=JV41AWY6016) |
| SB6 | 88.16 % | [KT380626.1](https://www.ncbi.nlm.nih.gov/nucleotide/KT380626.1?report=genbank&log$=nucltop&blast_rank=1&RID=JV3JHZDY014) |
| SB7 | 87.68 % | [KT380626.1](https://www.ncbi.nlm.nih.gov/nucleotide/KT380626.1?report=genbank&log$=nucltop&blast_rank=1&RID=JV4UHSSX016) |
| SB8 | 88.10 % | [KT380626.1](https://www.ncbi.nlm.nih.gov/nucleotide/KT380626.1?report=genbank&log$=nucltop&blast_rank=1&RID=JV4ZWW3T016) |
| SB9 | 88.29 % | [KT380626.1](https://www.ncbi.nlm.nih.gov/nucleotide/KT380626.1?report=genbank&log$=nucltop&blast_rank=1&RID=JV554F6D016) |
| SB10 | 88.10 % | [NC036104.1](https://www.ncbi.nlm.nih.gov/nucleotide/NC_036104.1?report=genbank&log$=nucltop&blast_rank=1&RID=JV5Z4S8B014) |
| SB11 | 88.28 % | [KT380626.1](https://www.ncbi.nlm.nih.gov/nucleotide/KT380626.1?report=genbank&log$=nucltop&blast_rank=1&RID=JV60RK9T016) |
| SB12 | 88.06 % | [KT380626.1](https://www.ncbi.nlm.nih.gov/nucleotide/KT380626.1?report=genbank&log$=nucltop&blast_rank=1&RID=JV6BGTXB014) |
| SB13 | 88.08 % | [KT380626.1](https://www.ncbi.nlm.nih.gov/nucleotide/KT380626.1?report=genbank&log$=nucltop&blast_rank=1&RID=JV6E6M7M016) |
| SB14 | 88.16 % | [KT380626.1](https://www.ncbi.nlm.nih.gov/nucleotide/KT380626.1?report=genbank&log$=nucltop&blast_rank=1&RID=JV8GTR2X016) |
| SB15 | 88.10 % | [KT380626.1](https://www.ncbi.nlm.nih.gov/nucleotide/KT380626.1?report=genbank&log$=nucltop&blast_rank=1&RID=JV6E6M7M016) |
| SB16 | 88.16 % | [KT380626.1](https://www.ncbi.nlm.nih.gov/nucleotide/KT380626.1?report=genbank&log$=nucltop&blast_rank=1&RID=JV7H7UKD014) |
| SB17 | 88.25 % | [KT380626.1](https://www.ncbi.nlm.nih.gov/nucleotide/KT380626.1?report=genbank&log$=nucltop&blast_rank=1&RID=JV7H7UKD014) |
| SB18 | 88.16 % | [KT380626.1](https://www.ncbi.nlm.nih.gov/nucleotide/KT380626.1?report=genbank&log$=nucltop&blast_rank=1&RID=JV7U3KH2014) |
| SB19 | 88.25 % | [KT380626.1](https://www.ncbi.nlm.nih.gov/nucleotide/KT380626.1?report=genbank&log$=nucltop&blast_rank=1&RID=JV7ZHEJ2016) |

**Table S6:** Mean and standard deviation values of body length and thoracic width of male and female stingless bees beetles

|  | **Length (mm)** | **Width (mm)** |
| --- | --- | --- |
| **Males** | 5.283 ± 0.435 | 2.308 ± 0.131 |
| **Females** | 5.148 ± 1.321 | 2.453 ± 0.347 |

**References**

Evans, Jay D, Jeffery S Pettis, and Hachiro Shimanuki. (2000) Mitochondrial DNA Relationships in an Emergent Pest of Honey Bees*: Aethina Tumida* (Coleoptera: Nitidulidae) from the United States and Africa. Ann. Entomol. Soc. Am, [https://doi.org/10.1603/0013-8746(2000)093[0415:MDRIAE]2.0.CO;2](https://doi.org/10.1603/0013-8746(2000)093%5b0415:MDRIAE%5d2.0.CO;2)

Evans, J. D., Spiewok, S., Teixeira, E. W., & Neumann, P. (2008) Microsatellite loci for the small hive beetle, *Aethina tumida*, a nest parasite of honey bees. Molecular Ecology Resources, <https://doi.org/10.1111/j.1471-8286.2007.02052.x>

Papach, A., Beaurepaire, A., Yañez, O., Huwiler, M., Williams, G. R., & Neumann, P. (2023) Multiple mating by both sexes in an invasive insect species, *Aethina tumida* (Coleoptera: Nitidulidae). Insect Science, <https://doi.org/10.1111/1744-7917.13112>

**Figure S1.** Ventral view of stingless bee beetles - male (left) and female (right)

**Figure S2.** Male genitalia of Underground Nest Beetle (UNB), *Aethina* sp.

**Figure S3.** Pygidium of Underground Nest Beetle (UNB), *Aethina* sp.

**Figure S4.** Pygidium of *Aethina tumida*

**Figure S5.** Phylogenetic tree inferred from from 65 sequences, 709bp dataset
